# Assessing Conservation Values: Biodiversity and Endemicity in Tropical Land Use Systems

**DOI:** 10.1371/journal.pone.0016238

**Published:** 2011-01-27

**Authors:** Matthias Waltert, Kadiri Serge Bobo, Stefanie Kaupa, Marcela Leija Montoya, Moses Sainge Nsanyi, Heleen Fermon

**Affiliations:** 1 Department of Conservation Biology, Georg-August-Universität Göttingen, Göttingen, Germany; 2 Department of Forestry, University of Dschang, Dschang, Cameroon; 3 Göttingen, Germany; 4 Facultad de Ciencias Biológicas de la Universidad Autónoma de Nuevo León, Nuevo León, México; 5 Botany Programme, Center for Tropical Forest Science (CTFS)/Korup Forest Dynamics Plot (KFDP), Department of Plant and Animal Sciences, Faculty of Science, University of Buea, Buea, Cameroon; Umea University, Sweden

## Abstract

Despite an increasing amount of data on the effects of tropical land use on continental forest fauna and flora, it is debatable whether the choice of the indicator variables allows for a proper evaluation of the role of modified habitats in mitigating the global biodiversity crisis. While many single-taxon studies have highlighted that species with narrow geographic ranges especially suffer from habitat modification, there is no multi-taxa study available which consistently focuses on geographic range composition of the studied indicator groups. We compiled geographic range data for 180 bird, 119 butterfly, 204 tree and 219 understorey plant species sampled along a gradient of habitat modification ranging from near-primary forest through young secondary forest and agroforestry systems to annual crops in the southwestern lowlands of Cameroon. We found very similar patterns of declining species richness with increasing habitat modification between taxon-specific groups of similar geographic range categories. At the 8 km^2^ spatial level, estimated richness of endemic species declined in all groups by 21% (birds) to 91% (trees) from forests to annual crops, while estimated richness of widespread species increased by +101% (trees) to +275% (understorey plants), or remained stable (- 2%, butterflies). Even traditional agroforestry systems lost estimated endemic species richness by - 18% (birds) to - 90% (understorey plants). Endemic species richness of one taxon explained between 37% and 57% of others (positive correlations) and taxon-specific richness in widespread species explained up to 76% of variation in richness of endemic species (negative correlations). The key implication of this study is that the range size aspect is fundamental in assessments of conservation value via species inventory data from modified habitats. The study also suggests that even ecologically friendly agricultural matrices may be of much lower value for tropical conservation than indicated by mere biodiversity value.

## Introduction

Since the seminal paper by Lawton *et al.* [1], numerous studies have dealt with biodiversity patterns of tropical land use gradients and analysed indicator properties of different taxa [2]–[5]. Looking more closely into these studies, it appears that patterns of alpha (point) diversity can be highly taxon-specific but that a general pattern of high beta turnover across habitats is visible in all taxa: most altered habitats usually also contain most altered biotic communities which may - or may not - be as diverse as primary forest but in any case composed of different species [6]. Although such inventory-based studies are being published and referred to in conservation journals, they more often allow conclusions rather on the ‘biodiversity value’ of modified landscapes rather than on their ‘conservation value’ [7]. E.g., while traditional agroforestry systems are often regarded as a potential quality matrix for the maintenance of diverse tropical forest biota [4], [8] and may thus appear as effectively contributing to global conservation, their biodiversity may in cases have little to do with the original rainforest biota. A number of single-taxon studies have however addressed more critically the comparative conservation value of agro-biodiversity by including degree of endemism/geographic range size [9]–[18] in the analyses, but we were unable to find a single multi-taxa land use gradient study which does so consistently for all taxa. We consider this lack of focus on conservation values as problematic since replacement of original assemblages by biota of different geographic range composition may have global conservation implications. We therefore advocate to more clearly separate ‘biodiversity value’ from ‘conservation value’ and to emphasise the latter issue much more in future studies. This should be especially important for multi-taxa studies which often receive a high number of citations, and which are most likely to influence landscape management. Here, using an existing dataset from southwestern Cameroon published elsewhere [13], [15], [16], we test the hypotheses that tropical deforestation and land use especially affect species of smaller geographic ranges and that agroecosystems favour richness of species of wider geographic ranges. We also hypothesized that the abundance of trees is a good predictor for the richness of biota of smaller geographic range categories. The dataset covers trees, understorey plants, fruit-feeding butterflies, and birds, sampled at 6 stations in each of four 8 km^2^ areas belonging to different habitat types (near-primary forest, young secondary forest, cocoa-agroforestry systems and annual crops). The taxa were chosen because they are frequently used indicators given the relatively moderate skills and sampling efforts needed for assessment [1]–[4], and because their geographic ranges are relatively well established and accessible [19]–[25] and Appendix S1.

## Results

### Birds

**Figure 1:**
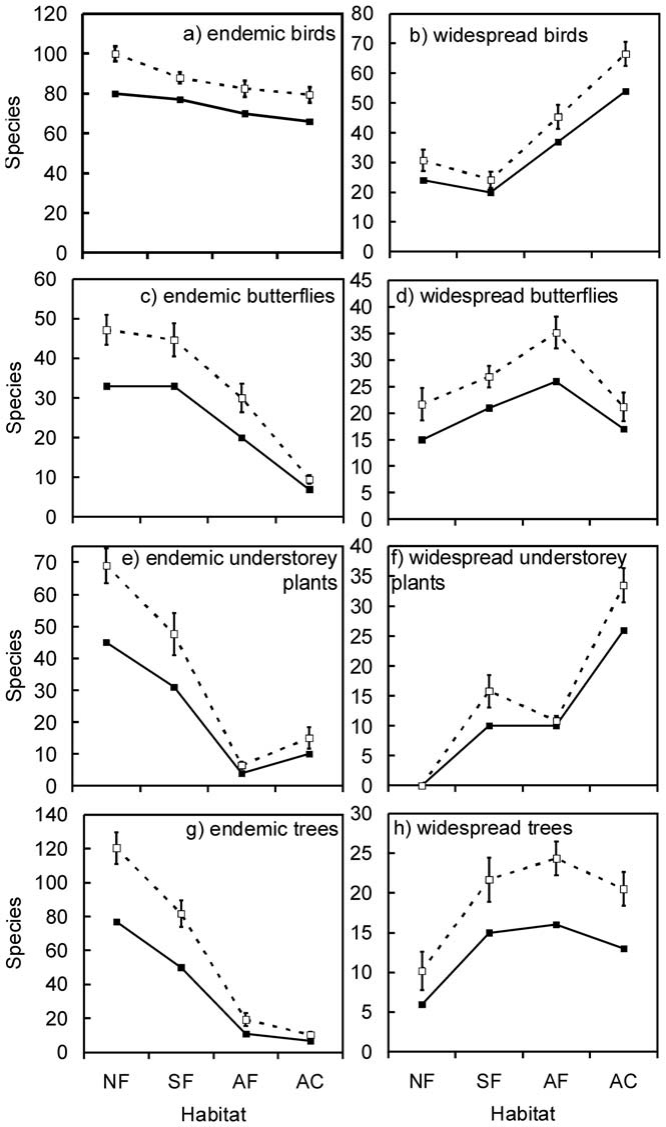
Species richness of different taxa, for two geographic range categories ‘endemic’ and ‘widespread’, at the 8 km^2^ spatial scale, for different habitats. Continuous lines represent observed (Sobs), dashed lines estimated species richness (Jackknife 1). Whiskers indicate ±1 SD. Habitats: NF, near primary forest; SF, secondary forest, AF, agroforestry systems; AC, annual crops.

Estimates of endemic bird species richness at sampling station (point) level were highest in, and did not differ significantly between (Tukey's Honest Significant Difference test, p>0.05), near-primary (NF, 64.2±4.1 S.D.) and secondary forest (SF, 64.9±5.3). They were however 27% lower in annual crops (AC: 47.1±12.9) and 21% lower in agroforestry systems (AF: 50.6±10.7) compared to NF (ANOVA, F_(3;20)_ = 6.28; p = 0.004 for estimated species richness). This pattern was very similar at habitat level (8 km^2^ scale), but with a slightly lower decrease (by 21%) between NF (100±3.7) and AC (79.3±4.0) (Figure 1a).

Point level estimates of widespread bird species richness showed an opposite trend in having lowest species richness in NF (19.7±4.2) and SF (15.7±1.8), but increasing by 88% to AC (37.1±7.5), and having intermediate richness (increase by 42%) in AF (27.9±5.3)(F _(3;20)_ = 20.48; p = 0.000). Estimates of widespread bird species richness did not differ significantly between NF and SF (Tukey's Honest Significant Difference test, p>0.05). Again, this pattern was similar at habitat level, where the increase from NF (30.7±3.8) to AC (66.5±5.6) amounted to 117%, and 48% to AF (45.3±2.8) (Figure 1b).

### Butterflies

Point level estimates of endemic butterfly species richness were similar in NF (19.5±6.3) and SF (21.0±3.6) but declined by 79% from NF to AC (4.1±2.2), and by 46% to AF (10.5±4.6)(F_(3;20)_ = 24.78; p = 0.000 for observed, F _(3;20)_ = 20,0527; p = 0.000 for estimated richness). This pattern was almost identical at habitat level, with an 80% decrease from NF (47.2±3.8) to AC (9.5±1.1) and a 36% decrease from NF to AF (30.0±3.7) (Figure 1c).

In contrast, point estimates of widespread butterfly species richness increased from NF (8.1±3.8) towards AF (17.0±5.2) by 110%, but were again lower in AC (10.7±3.0), at similar levels to NF (F _(3;20)_ = 6.82; p = 0.002). Again, this pattern was similar at habitat level, with a 62% increase only between NF (21.7±3.1) and AF (35.2±3.0), and values of AC (21.2±2.7) very similar to NF (Figure 1d).

### Understory plants

Point level estimates of endemic understorey plant species richness dropped by 91% from NF (18.2±5.1) to AF (1.6±1.4) and were similarly low in AC (4.1±3.5), representing a decrease of 77% compared to NF (F _(3;20)_ = 22.18; p = 0.000). Estimates at habitat level followed an identical pattern, with a decrease of 88% from NF (69.0±5.32) to AF (6.5±1.1) and of 71% to AC (15.0±3.4) (Figure 1e).

In contrast, widespread understorey plant species richness increased at point level from 0.0 (±0.0) species in NF to 15.4 (±4.3) species in AC, with an average of 10.8 (±0.8) species in AF. The percentage of increase from SF (4.1±3.3) to AC (15.8±2.7 species) amounted to 275%, but was only 43% to from SF to AF (7.2±1.6) (F_(3;20)_ = 32.68; p = 0.000). At habitat level, a similar pattern was found but with relatively higher values for SF (15.8±2.7) and a proportional increase from SF to AC (33.5±2.8) of 112% and of 32% to AF (10.8±0.8) (Figure 1f).

### Trees

Point estimates of endemic tree species richness dropped steadily from an estimated 31.1±9.5 species in NF, by 90% to 3.2±1.5 species in AC (F _(3;20)_ = 23.34; p = 0.000). In SF (19.6±9.6), the decrease from NF amounted to 37%. AF sampling stations (3.2±2.7) had similar values to AC, loosing 84% of species compared to SF. At habitat level, the pattern was again identical: the loss of estimated endemic tree species richness amounted to 91% between NF (120.3±9.3) and AC (10.3±1.7), the drop being still 32% from NF to SF (81.7±7.9), and still 76% from SF to AF (19.3±3.8) (Figure 1g).

Point estimates of widespread tree species richness were very low in NF (1.6±1.8), higher at SF and AC (6.7±3.9, 5.4±2.0, respectively), reaching 8.2±3.7 species in AF sampling stations (F _(3;20)_ = 5.28; p = 0.008). At habitat level, estimates of widespread tree species richness doubled from NF (10.2±2.4) to SF (21.7±2.8) and AC (20.5±2.1), representing an increase by 101%, and was even higher in AF (24.3±2.1) (Figure 1h).

### Correlations

There were strong positive correlations between endemic species richness of one taxon/group and endemic species richness of others, explaining between 37 and 56% (R^2^) of the variation (Spearman Rank correlation coefficients R amounted to between 0.61 and 0.75, *P*≤0.001). We also found strong positive correlations between widespread bird species richness and that of widespread understorey plants, as well as between widespread butterfly species richness and widespread tree species richness (Table 1).

**Table 1:** Spearman Rank correlation coefficients R for relationships between estimated (first-order Jackknife) species richness of geographic range groups of different taxa.

Endemics vs Endemics (n = 24 sampling stations in all cases)				
	Butterflies	Understorey Plants	Trees	
Birds	**0.68**	**0.62**	**0.61**	
	***P*** **<0.001**	***P*** ** = 0.001**	***P*** ** = 0.001**	
Butterflies		**0.61**	**0.71**	
		***P*** ** = 0.001**	***P*** **<0.001**	
Understorey Plants			**0.75**	
			***P*** **<0.001**	

Widespread vs Widespread (n = 24 in all cases)				
	Butterflies	Understorey Plants	Trees	
Birds	−0.02	**0.73**	0.22	
	*P* = 0.93	***P*** **<0.001**	*P* = 0.30	
Butterflies		0.21	**0.65**	
		*P* = 0.34	***P*** **<0.001**	
Understorey Plants			0.37	
			*P* = 0.08	

Endemics vs Widespread (n = 24 sampling stations in all cases)				
	Endemic Birds	Endemic Butterflies	Endemic Understorey Plants	Endemic Trees
Widespread Birds	**−0.67**	**−0.87**	**−0.56**	**−0.71**
	***P*** **<0.001**	***P*** **<0.001**	***P*** ** = 0.004**	***P*** **<0.001**
Widespread Butterflies		−0.05	**−0.51**	−0.25
		*P* = 0.82	***P*** ** = 0.01**	*P* = 0.24
Widespread Understorey Plants			**−0.68**	**−0.77**
			***P*** **<0.001**	***P*** **<0.001**
Widespread Trees				**−0.41**
				***P*** ** = 0.04**

Significant (*P*<0.05) values in bold.

Likewise, in most groups/taxa there were strong negative correlations between widespread species richness and endemic species richness (Spearman Rank corrleation coefficients R between -0.41 and – 0.87, P<0.05).

There were also strong correlations between overall tree abundance and endemic species richness of birds (Spearman R = 0.65, P<0.001), butterflies (R = 0.69, P<0.001) and trees (R = 0.78, P<0.001) and a moderately strong correlation between overall tree abundance and endemic understorey plant species richness (R = 0.60, P = 0.002).

## Discussion

Our study, based on assessments at the 8 km^2^-level, revealed that endemics of major indicator taxa show a steady decline in richness with increasing forest conversion, i.e. from near-primary forest to annual crops. In contrast, richness in widespread birds, understorey plants and trees increased along this gradient. Richness of widespread butterfly species also increased in secondary forest and agroforestry systems but reached again near-primary forest levels in annual crops.

If analyses at these spatial scales provide an indication of biodiversity patterns at the regional level, there may be important implications for the design and analysis of environmental impact assessments, as well as for global conservation strategies. The key conclusion is that endemic species richness is a potentially powerful indicator for conservation evaluation of modified habitats. One example: agroforestry systems have been found to maintain substantial levels of biodiversity and have therefore been largely appraised as an all too perfect fusion of economic yield and nature conservation [26], [27]. Based on the results of our own approach, however, we may argue that this seemingly ideal land use form is prone to being overrated regarding its conservation value. Some of the potential reasons for these euphemistic appraisals are study biases towards investigating mere species richness and abundance, and only occasionally community composition [28] and beta diversity between primary- and agro-forests [6]. Furthermore, agroforestry study sites are often situated close to primary or secondary forests, facilitating influx of mobile organisms into these systems [29] and thereby leading to overestimation of both biodiversity and conservation value of these modified systems.

Reviewing studies which compare biodiversity value of primary forests and agroforestry systems, Scales & Marsden [30] report reduced species richness in modified agroforests in 34 out of 43 studies. In some cases, declines along land use gradients may be so gradual that there seems to be no problem to rank agroforestry systems close to natural and secondary forests. Among the studies listed [30], there are also several which indicate higher or similar species richness in agroforestry systems compared to forest, even for vertebrates [31]. However, while such studies tell us something about functional diversity and ecology of modified systems, they are of little help when they are put into the context of the global biodiversity crisis. Our study differs from such work in that it explicitly addresses conservation values of land use systems based on several indicator groups. It largely confirms what earlier single taxon studies [13], [15] indicated: namely that forest modification and land use affect endemic species of different indicator taxa in a very similar way, reflecting that species turnover from forest to farmland is to a large extent a replacement of endemic by widespread species. While ecological requirements of species of narrow geographic ranges are often little known, our results suggest this reflects to a large extent the change in tree abundance and species richness, the two parameters which were both correlated moderately to strongly with endemic species richness. However, there is still much to be learned about the ecology associated with species of narrow geographic ranges, and future studies should aim at exploring the causal relationships between endemic species declines and associated biotic and abiotic environment.

We advocate a pre-cautionary approach when putting biodiversity data from the tropical agricultural matrix into the context of conservation evaluation. We note that the current knowledge on the conservation benefits of tropical land use systems is still limited and that research at different spatial scales is still urgently needed. Given this state of knowledge, we suggest it is preferable to invest the limited funds for conservation of wet tropical forest region biodiversity into the proper protection and management of remaining natural forests.

### Study area

The study was carried out within an appr. 40 km^2^ section of the Support Zone (SZ) of Korup National Park, in the South-western region of Cameroon [16]. This region is part of the Guineo-Congolian forest [32] and also part of the Hygrophylous Coastal Evergreen Rainforest which occurs along the Gulf of Biafra within the Cross-Sanaga-Bioko Coastal Forest ecoregion [32], [33]. This ecoregion is considered an important center of plant diversity because of its probable isolation during the Pleistocene [34] and holds an assemblage of endemic primates known as the Cameroon faunal group [35]–[36]. The region is also exceptionally rich in butterflies [37] and birds [38].

The studied sampling stations were all situated in the populated part of the SZ, where farming is restricted to the immediate surroundings of the villages, leaving most of the area forested. The land use types chosen represent different forms of common land use practice, and are situated along a gradient of human disturbance where near-primary forest (NF) serves as a reference. They basically differ in two important characteristics of habitats: Habitat complexity referring to the vertical structure of vegetation and habitat heterogeneity expressed in the horizontal variation of the habitat's features. All sites outside the near-primary forest, i.e. secondary forest (SF), agroforestry systems (AF) and annual crop farms (AC), are located at the vicinity of the forest edge. The main characteristics of the chosen habitats are as follows [15], [16]:

1. NF: wet evergreen forest with high tree species richness. Closed canopy averages 35–45 m. The dominant trees are *Oubangia alata* and *Gilbertiodendron demonstrans*.
2. SF: moist evergreen forest which has been cleared for farming along roads about 15 years ago. These forests have a relatively closed canopy. Canopy height averages 25–30 m. Characteristic trees are *Elaeis guineensis, Barteria fistulosa, Rauvolfia vomitoria* and *Pycnanthus angolensis.*
3. CF: cocoa/coffee plantations shaded by natural forest trees of up to 25 m height. Apart from *Theobroma cacao* (Cocoa) and *Coffea robusta* (Coffee) trees, remnant *Elaeis guineensis* (Oil palm) and *Dacryodes edulis* (Plum) trees are characteristic.
4. AC: open monoculture of manioc, remnant forest trees, remnant oil palms, no planted shade trees, dead wood, *Chromolaena odorata* and farm bush thickets; it is a dynamic habitat, due to the short cycles of the cultivated plants and associated human activities.

## Methods

### Data collection

Six sampling stations were selected in each of the above mentioned habitats, adding up to a total of 24 stations located at least 500 m apart from each other and covering an approximate area of ca. 8 km^2^ in each habitat. Topographically, all study sites were situated at an altitude of about 250 m above sea level. For vegetation (tree and understorey plant) sampling, centred on each sampling station, plots of 50 m ×50 m were established. Each plot was divided into nine subplots of 10 m ×10 m (one subplot in the centre and eight others at the borders) so as to have 10 m in between subplots and spreading over 2,500 m^2^ in total at each study site.

In each 10 m ×10 m subplot, a 1 m ×1 m small plot, established in its centre, was used to collect data for understorey plants. Understorey plants were defined as all vascular plants of less than 1.3 m height, and (overstorey) trees as all trees of more than 10 cm in diameter at 1.3 m height (DBH). In total, data were organised in 216 understorey plant samples (9 small plots ×24 sampling stations), as well as 216 tree samples (9 subplots ×24 sampling stations). In total, 1,230 understorey plant and 856 tree individuals were recorded and identified at least to morphospecies level. Species of uncertain identity were not assigned to geographic range categories. Therefore, of the original dataset containing 350 understorey plant and 226 tree morphospecies, we used 219 (63%) and 204 (90%) for our analyses, respectively.

Fruit-feeding butterfly data were collected within 50 m from the centre of each sampling station during the dry season, between 27 December 2003 and 10 March 2004. We used three cylindrical gauze-traps [39]–[41] baited with rotten bananas. These three traps were installed at about 1.5 m above the soil surface and controlled daily for nine days at each sampling station. Specimens collected on one sampling day in the three traps were pooled per sampling station, resulting in a total of 216 butterfly samples (24 sampling stations ×9 days). A total of 1,167 butterfly individuals of 119 species were collected, labelled and later identified using D'Abrera [19], [20], Hecq [21] and Larsen [22]. All individuals were identified to species level.

Bird surveys were carried out between 23 December 2003 and 5 March 2004 using point counts of birds within a range of 50 m from the centre of the sampling station. As most land use systems (AF, AC) were only of small size (<2 ha), small-scale point counting was the only possible method, regardless of the fact that bird point diversity in tropical forests may only reflect a small proportion of the overall alpha-diversity [42]. All visits were conducted between 6:00 and 9:00 am for 20 min, and both visual and acoustical detections were recorded. Fieldwork was done by the same observer throughout the survey and sites were visited nine times, respectively. A total of 4,530 records of 180 species were obtained and identified mainly with Borrow and Demey [23], but also Brown *et al.* [24] and Keith *et al.* [25].

### Data analysis

We reviewed available geographic range information for all taxa and clustered species accordingly, resulting in groups of ‘endemic” and ‘widespread” species (Table 2). A third category of ‘medium distributional range” was introduced for butterflies and plants in order to obtain comparable percentages of endemic species across the four taxa. Hence, for butterflies 16 species, for understorey plants 50 spp. and for trees 61 species were omitted from the analyses.

**Table 2:** Geographic range descriptions for range size codes of endemic species, those with medium-sized ranges and widespread species, for birds, butterflies and plants, separately.

	Birds			Butterflies			Understorey Plants/Trees		
		Range	# spp.		Range	# spp.		Range	#/# spp.
**Endemic**	**1**	Cameroon-Gabon lowlands restricted	1	**1**	endemic to eastern Nigeria and southwestern Cameroon	2	**1**	Endemic to SW Cameroon/SE Nigeria border	14/24
				**2**	from Nigeria to the Cameroon-Gabon-Congo zone	20	**2**	From SW Cameroon/SE Nigeria border to W Benin/Togo border **OR** from SW Cameroon/SE Nigeria border to Gabon, Congo, Equatorial Guinea, including Fernando Po and Sao Tome and Principe islands.	44/54
				**3**	from Nigeria to D.R. Congo or DRC-Uganda border	26	**3**	From SW Cameroon/SE Nigeria, Gabon, Congo, Equatorial Guinea, including Fernando Po and Sao Tome and Principe islands to W Ivory Coast **OR** from SW Cameroon/SE Nigeria, Gabon, Congo, Equatorial Guinea to DRC/Uganda border and Angola	22/33
	**2**	Guineo-Congolian Forest Biome restricted	115	**4**	from Nigeria to east of Rift Valley	9			
**Medium distributional range**				**5**	from western West Africa to the Nigeria-Cameroon border	6	**4**	From Benin to DRC/Uganda border and Angola OR from SW Cameroon/SE Nigeria, Gabon, Congo, Equatorial Guinea to Sierra Leone OR from SW Cameroon/SE Nigeria, Gabon, Congo, Equatorial Guinea to Kenya and Angola	26/25
				**6**	from western West Africa to the Cameroon-Gabon-Congo zone	10	**5**	From Sierra Leone to D.R. Congo/Uganda, and Angola OR from SW Cameroon/SE Nigeria, Gabon, Congo, Equatorial Guinea to Senegambia OR from SW Cameroon/SE Nigeria, Gabon, Congo, Equatorial Guinea to E Rift Valley, and Angola	13/26
							**6**	From Senegambia to D.R. Congo/Uganda, and Angola	11/12
**Widespread**	**3**	African Rainforest	2	**7**	from western West Africa to D.R. Congo or DRC-Uganda border	14			
				**8**	from western West Africa to Uganda or Western Kenya	19	**7**	From Senegambia to Tanzania and Angola OR From Ivory Coast to Sudan, East Africa/Mozambique	5/3
				**9**	from western West Africa to east of Rift Valley	3	**8**	From Senegambia to Sudan, East Africa/Mozambique, and Angola	12/11
	**4**	Ubiquitous in Africa	62	**10**	found throughout Africa in suitable habitats	10	**9**	Throughout tropical Africa in suitable habitats	14/16

Also given are numbers of species in each category.

For bird distribution data, we used Fishpool and Evans [43] who classified 116 of our study species as being restricted to the Guinea-Congolian forest biome [33], spanning from east Guinea to west D.R. Congo and southwards to Congo and Gabon. This group of 116 bird species was categorised as ‘endemic’. The remaining 64 species can be classified as non-biome-restricted [43], and were thus categorised as ‘widespread”. This categorisation is also followed in an earlier publication [16].

For butterfly distribution data, we followed Larsen [22] to obtain geographic range information for butterflies, dividing them into ten range size categories, from 1 - most range-restricted to 10 -most widespread; 57 of the 119 species belonged to categories 1 to 4, with geographic ranges roughly situated within the Guinea-Congolian forest biome, and were categorised as ‘endemic”. Those with categories 7 to 10 were grouped into the ‘widespread” category, which included 46 species.

We obtained understorey plant and tree geographic ranges from the Global Biodiversity Information Facility, Aubréville *et al.*, Hutchinson & Dalziel, and several volumes of the Flora of Tropical East Africa (Appendix S1). As in butterflies and birds, we categorised geographic ranges ranging from 1 to 9, with ‘endemic” species spanning at the most from southwest Cameroon to West Ivory Coast or eastwards to the D.R. Congo- Uganda border (categories 1–3). ”Widespread” species were defined as those of the categories 7 to 9 with a geographic range size of at least the magnitude of the area from Senegambia to Tanzania and Angola. Eighty of the 219 understorey plant species and 111 of 204 tree species were thus defined as ‘endemic”, whereas 31 understorey plants and 30 tree species were classified as ‘widespread”. For 48 understorey plant species geographic range size data could not be procured and they were thus excluded from further analysis.

As biodiversity field studies in the tropics usually fail to record all of the present species [44] we calculated an ‘estimated’ species richness in order to gain a more accurate picture of the actual species richness. Such calculations of estimated species richness take into account the frequency patterns of the ‘observed’, species. We used the first-order jackknife method initially designed to estimate population size from capture to recapture data, allowing capture probabilities to vary by individuals [45]. This model can equally be applied to estimations of species richness [46]–[51]. Calculations of estimated species richness were made using EstimateS Win 7.5.0 and 8.2.0 by Colwell [52] using 200 randomizations.

Calculations of observed and estimated species richness were carried out for both point species richness, based on data of nine spatial subplots per point, spread over 2,500 m^2^ (trees, understorey plants) and nine temporal subsamples which covered a similar circular area (butterflies, birds), but also at the 8 km^2^ habitat level, based on data from the six sampling stations in each habitat. One-way ANOVA was done to detect responses to habitat variation for each group of geographic range category. Spearman rank correlation coefficients were established with STATISTICA V.9 (StatSoft) to illustrate the relationships between the estimated species richness of taxa/geographic range groups and vegetation parameters.

## Supporting Information

Appendix S1 Literature sources for plant geographic ranges.
(DOC)
