# Supplementary material for: Assessing Conservation Values: Biodiversity and Endemicity in Tropical Land Use Systems
Source: PLoS One. 2011 Jan 27;6(1):e16238. doi: 10.1371/journal.pone.0016238 (PMC3029302; doi:10.1371/journal.pone.0016238)
Supplement: Appendix S1 — Literature sources for plant geographic ranges. (DOC) [file pone.0016238.s001.doc]

**Appendix S1: Literature sources for plant geographic ranges**

1. Global Biodiversity Information Facility (2010). GBIF Data Portal. Available: [http://www.gbif.org](http://www.gbif.org/). Accessed 2010 Dec 31.

2. Aubréville A, Leroy JF, Morat P, Satabie B, Achoundong G. et al. (1970–1984) Flore du Cameroun Vols. 9, 11, 15, 27. Paris and Yaoundé: Muséum National d’ Histoire Naturelle, Paris and Ministère de la Récherche Scientifique et Technique, Yaoundé, Cameroun.

3. Hutchinson J, Dalziel JM, Hepper FN (1954–1972) Flora Of West Tropical Africa Vols. 1–3, 2nd edn. London: Crown Agents.

4. Beentje H, Smith SAL (2000) Flora of tropical East Africa: Compositae Part 1. Rotterdam: Balkema.

5. Beentje H (2002) Flora of tropical East Africa: Compositae Part 2. Rotterdam: Balkema.

6. Bridson DM, Beentje H, Polhill RM (1988) Flora of tropical East Africa: Rubiaceae Part 2. Rotterdam: Balkema.

7. Carter S, Beentje H, Polhill RM (1988) Flora of tropical East Africa: Euphorbiaceae Part 2. Rotterdam: Balkema.

8. Gillet JB, Beentje H, Polhill RM (1991) Flora of tropical East Africa: Burseraceae. Rotterdam: Balkema.

9. Grey-Wilson C, Beentje H, Polhill RM (1986) Flora of tropical East Africa: Violaceae. Rotterdam: Rotterdam.

10. Hemsley JH, Turril WB (1956) Flora of tropical East Africa: Connaraceae. Rotterdam: Balkema.

11. Lock JM, Beentje H, Polhill RM (1993) Flora of tropical East Africa: Musaceae. Rotterdam: Balkema.

12. Omino EA (2002) Flora of tropical East Africa: Apocynaceae Part 1. Rotterdam: Balkema.

13. Polhill RM, Hubbard CE (1966) Flora of tropical East Africa: Ulmaceae. Rotterdam: Balkema.

14. Smith AR, Beentje H, Polhill RM (1987) Flora of tropical East Africa: Euphorbiaceae Part 1. Rotterdam: Balkema.

15. Styles BT, White F, Beentje H, Polhill RM (1991) Flora of tropical East Africa: Meliaceae. Rotterdam: Balkema.

16. Verdcourt B, Beentje H, Milne-Redhead E (1971) Flora of tropical East Africa: Annonaceae. Rotterdam: Balkema.

17. Verdcourt B, Beentje H, Polhill RM (1976) Flora of tropical East Africa: Rubiaceae Part 1. Rotterdam: Balkema.

18. Verdcourt B, Beentje H, Polhill RM (1991) Flora of tropical East Africa: Rubiaceae Part 3. Rotterdam: Balkema.

19. Verdcourt B, Beentje H (2001) Flora of tropical East Africa: Myrtaceae. Rotterdam: Balkema.

20. Wickens GE, Beentje H, Polhill RM (1973) Flora of tropical East Africa: Combretaceae. Rotterdam: Balkema.
